# MMP-15 Is Upregulated in Preeclampsia, but Does Not Cleave Endoglin to Produce Soluble Endoglin

**DOI:** 10.1371/journal.pone.0039864

**Published:** 2012-06-29

**Authors:** Tu’uhevaha J. Kaitu’u-Lino, Kirsten Palmer, Laura Tuohey, Louie Ye, Stephen Tong

**Affiliations:** The Translational Obstetrics Group, The Department of Obstetrics and Gynaecology, Mercy Hospital for Women, University of Melbourne, Heidelberg, Victoria, Australia; Otto-von-Guericke University Magdeburg Medical Faculty, Germany

## Abstract

Preeclampsia is a major pregnancy complication, characterized by severe endothelial dysfunction, hypertension and maternal end-organ damage. Soluble endoglin is an anti-angiogenic protein released from placenta and thought to play a central role in causing the endothelial dysfunction and maternal organ injury seen in severe preeclampsia. We recently reported MMP-14 was the protease producing placentally-derived soluble endoglin by cleaving full-length endoglin present on the syncytiotrophoblast surface. This find identifies a specific drug target for severe preeclampsia; interfering with MMP-14 mediated cleavage of endoglin could decrease soluble endoglin production, ameliorating clinical disease. However, experimental MMP-14 inhibition alone only partially repressed soluble endoglin production, implying other proteases might have a role in producing soluble endoglin. Here we investigated whether MMP-15–phylogenetically the closest MMP relative to MMP-14 with 66% sequence similarity–also cleaves endoglin to produce soluble endoglin. MMP-15 was localized to the syncytiotrophoblast layer of the placenta, the same site where endoglin was localized. Interestingly, it was significantly (p = 0.03) up-regulated in placentas from severe early-onset preeclamptic pregnancies (n = 8) compared to gestationally matched preterm controls (n = 8). However, siRNA knockdown of MMP-15 yielded no significant decrease of soluble endoglin production from either HUVECs or syncytialised BeWo cells *in vitro*. Importantly, concurrent siRNA knockdown of both MMP-14 and MMP-15 in HUVECS did not yield further decrease in soluble endoglin production compared to MMP-14 siRNA alone. We conclude MMP-15 is up-regulated in preeclampsia, but does not cleave endoglin to produce soluble endoglin.

## Introduction

Preeclampsia affects 3–5% of pregnancies and is a leading cause of maternal and perinatal mortality and morbidity. [1], [2] Delivery of the baby and placenta is the only current cure; however if the pregnancy is considerably preterm, this inflicts severe prematurity on the baby.

A significant advance in the understanding of its pathogenesis was made with the characterization of two anti-angiogenic proteins - soluble fms-like tyrosine kinase-1 (sFlt-1) and soluble endoglin (sEng) - as the likely factors released from preeclamptic placentas causing endothelial dysfunction and maternal organ injury. Both are significantly up-regulated in the sera of women with preeclampsia with levels correlating with clinical disease severity. Adenoviral administration of sFlt-1 to rats induces hypertension and proteinuria, hallmarks of preeclampsia. Importantly, co-administration of both sFlt-1 and sEng to rats recapitulates the full spectrum of end-organ injury seen in severe preeclampsia (severe proteinuria, low platelets, deranged liver function tests and cerebral oedema). [3], [4], [5].

The characterisation of these two anti-angiogenic factors in preeclampsia is significant as they represent targets to develop therapeutics. A drug that either decreases the production or biological activity of either factor could possibly quench the disease. If developed, such a drug would be an important advance in the management of this disease.

Full-length membrane bound endoglin is a co-receptor for transforming growth factor-β (TGF-β), and is highly expressed on both endothelial cells and the syncytiotrophoblast. [6] MMP-14, also known as MT1-MMP is one of six membrane-type MMPs, and has been widely studied for its role in cancer progression and metastasis. [7], [8], [9], [10] Through over-expression systems in COS-7 cells, Hawinkels et al showed MMP-14 was the cleavage protease of membrane bound endoglin, producing soluble endoglin that is then released. [11] Through mutagenesis experiments, they mapped the cleavage point on membrane bound endoglin to a specific glycine-leucine point near the transmembrane domain region.

We subsequently reported MMP-14 was highly expressed in placenta, co-localised with endoglin, and cleaves endoglin to produce soluble endoglin in placental cells [12]. Our data strongly suggests membrane bound MMP-14 was responsible for producing soluble endoglin in preeclampsia.

**Table 1:** Clinical Characteristics of the preeclamptic cohort.

	Preterm Control (n = 8)	Preeclampsia (n = 8)
**Maternal Age** Mean years (±SEM)	31.0 (1.2)	32.6 (1.6)
**Gestation at Delivery** Mean weeks (±SEM)	30.9 (0.9)	32.2 (1.3)
**Ethinicity**–No. (%) Caucasian Asian	7 (87.5) 1(12.5)	5 (62.5) 3 (37.5)
**BMI (kg/m^2^)** Mean (±SEM)	30.9 (1.7)	26.4 (1.7)
**Primiparous** No. (%)	2 (25)	5 (62.5)
**SBP at Delivery** Mean mmHg (±SEM)	121.0 (3.3)	172.1 (5.8)**
**DBP at Delivery** Mean mmHg (±SEM)	73.4 (3.1)	110.7 (4.7)**
**Birthweight** Mean grams (±SEM)	1622 (176.4)	1551 (268.3)

Shown are clinical details of the two cohorts from whom we obtained placentas for our analyses. The preeclamptic cohort all had severe preeclampsia necessitating delivery preterm. Preterm controls where those who were delivered early for other indications but did not have preeclampsia. **p<0.001. SEM =  standard error of the mean, SBP =  systolic blood pressure, DBP =  diastolic blood pressure, BMI =  body mass index and GA =  gestational age.

However, we noted inhibition of MMP-14 only partially repressed soluble endoglin production (∼50%), implying other protease(s) might also have a role in soluble endoglin production. We have therefore sought to characterise these other proteases and identified MMP-15 as a strong candidate for the following reasons: 1) it is phylogenetically the most closely related to MMP-14 of all MMPs with 66% homology at the catalytic domains [13], 2) *in silico* analysis of MMP15 suggests it is expressed >30 fold expression in placenta relative to average expression in other tissues [14], 3) it has an interchangeable role with MMP-14 in facilitating placental development in mice, implying shared roles in placental biology [15]. Therefore, we examined the expression of MMP-15 in preeclamptic placentas, localized its expression, and investigated whether it cleaves endoglin to produce soluble endoglin.

## Materials and Methods

### Tissue Collection

Women presenting to two tertiary women’s hospitals in Melbourne, Australia, between 2008–2009 gave informed written consent for placental tissue collection. Placenta was obtained from preterm pregnancies not complicated by preeclampsia (n = 8) and those complicated by severe early-onset preeclampsia (n = 8). Severe preeclamptics were diagnosed in accordance with ACOG guidelines and included the presence of hypertension >160/110 on two occasions greater than 6 hours apart, proteinuria >5 g/day, oliguria <500 ml/day, visual disturbance, pulmonary oedema, right upper quadrant pain, abnormal liver function, thrombocytopenia or fetal growth restriction [16]. In addition, all samples were obtained from cases of early-onset preterm pre-eclampsia, defined as requiring delivery <34 weeks gestation. Pre-term control placentas were selected from women presenting with pre-term rupture of membranes or spontaneous preterm labor without evidence of infection (histopathological examination of the placentas), hypertensive disease or maternal co-morbidities. Patient characteristics are outlined in table 1.

**Figure 1:**
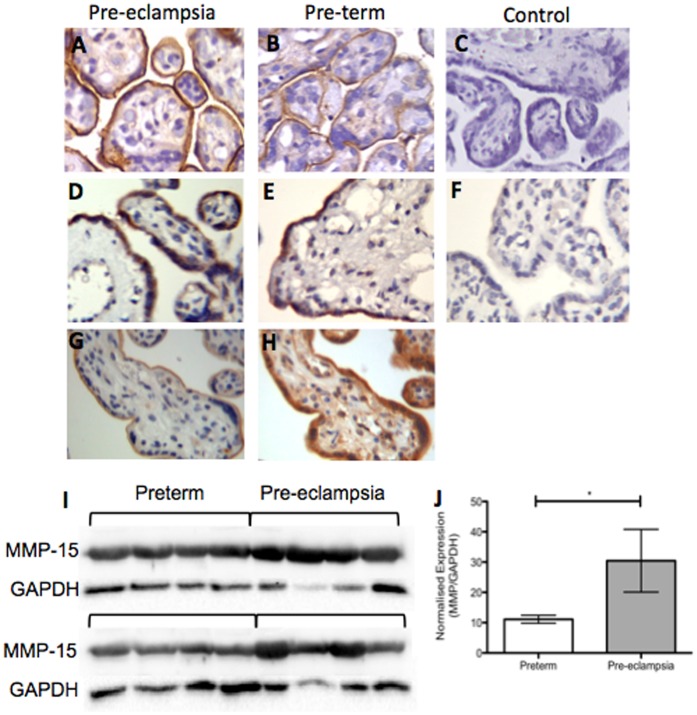
MMP-15 is localised to the syncytiotrophoblast and up-regulated in preeclamptic placenta. Representative immunohistochemistry for endoglin (**A, B**) and MMP-15 (**D, E**), shows both proteins localize to the syncytiotrophoblast in pre-eclamptic (**A, D**) and pre-term control (**B, E**) placentas. Immunohistochemistry on serial sections (2 µm) of placenta revealed co-localisation of endoglin (**G**) and MMP-15 (**H**) to the syncytiotrophoblast. No staining was observed in isotype controls (**C, F**). Densitometric analysis of western blots for MMP-15 (**I, J**) revealed a significant increase in preeclamptic placentas (n = 8) compared to pre-term controls (n = 8). *p≤0.05.

Placental tissue was obtained immediately following delivery. Placental tissue (excluding fetal membranes) was removed and washed briefly in sterile phosphate-buffered saline (PBS). Samples for protein extraction were frozen within 15 minutes of delivery and stored at −80°C. A portion of each placenta was also fixed in 10% buffered formalin for histology.

Human Ethics approval was obtained for this study from both the Southern Health Human Research Ethics Committee and the Mercy Health Human Research Ethics Committee.

### Western Blot Analysis and ELISAs

20 µg of placental lysates were separated on 10% polyacrylamide gels with wet transfer to PVDF membranes (Millipore, Billerica, MA). Membranes were blocked prior to blotting overnight with an antibody targeting MMP-15 (1∶1000, Millipore, Billerica, MA) or GAPDH (1∶5000, Cell Signalling Technology, Danvers, MA). Membranes were then visualized using an enhanced chemiluminescence detection system (Santa Cruz Biotechnology) and ChemiDoc XRS (BioRad, Hercules, CA). GAPDH was used as a loading control. Relative densitometry was determined using Image Lab (BioRad).

Soluble endoglin levels were measured in conditioned culture media using the human endoglin ELISA (R&D systems according to manufacturer’s instructions. Optical density was determined using a BioRad X-Mark microplate spectrophotometer (BioRad) and endoglin levels determined using BioRad Microplate manager 6 software.

### Immunohistochemistry

Endoglin and MMP-15 immunhistochemistry was conducted on placental tissue collected from either pre-eclamptic or pre-term control pregnancies. Paraffin sections (5 µm) of formalin-fixed tissues were dewaxed in Xylene and rehydrated through descending grades of ethanol. Sections were then heated for 20 min on defrost in a 700-W microwave, followed by cooling to room temperature (RT) for 30 mins. They were washed for 10 min in Phosphate-buffered saline pH 7.6 (PBS), and immersed in 3% H_2_O_2_ in methanol for 10 min at RT. Sections were then washed with PBS, before immersion into Dako blocking buffer (DAKO) for 10 mins and incubated for 1 h at RT with mouse anti human MMP-15 (R&D systems, MN, USA) at 25 µg/ml in 1%BSA/PBS. For Isotype controls, primary antibody was substituted with mouse IgG. The SuperPicTure kit (Invitrogen, Carlsbad, CA) was the applied according to manufacturer’s instructions to reveal the MMP-15 staining. Sections were lightly counterstained with Harris hematoxylin (Accustain; Sigma Diagnostics, Castle Hill, NSW, Australia), dehydrated, and mounted using DPX mounting medium (BDH Laboratory Supplies, Poole, England). For co-localisation studies, serial sections (2 µm) were stained in the same manner as described above.

**Figure 2:**
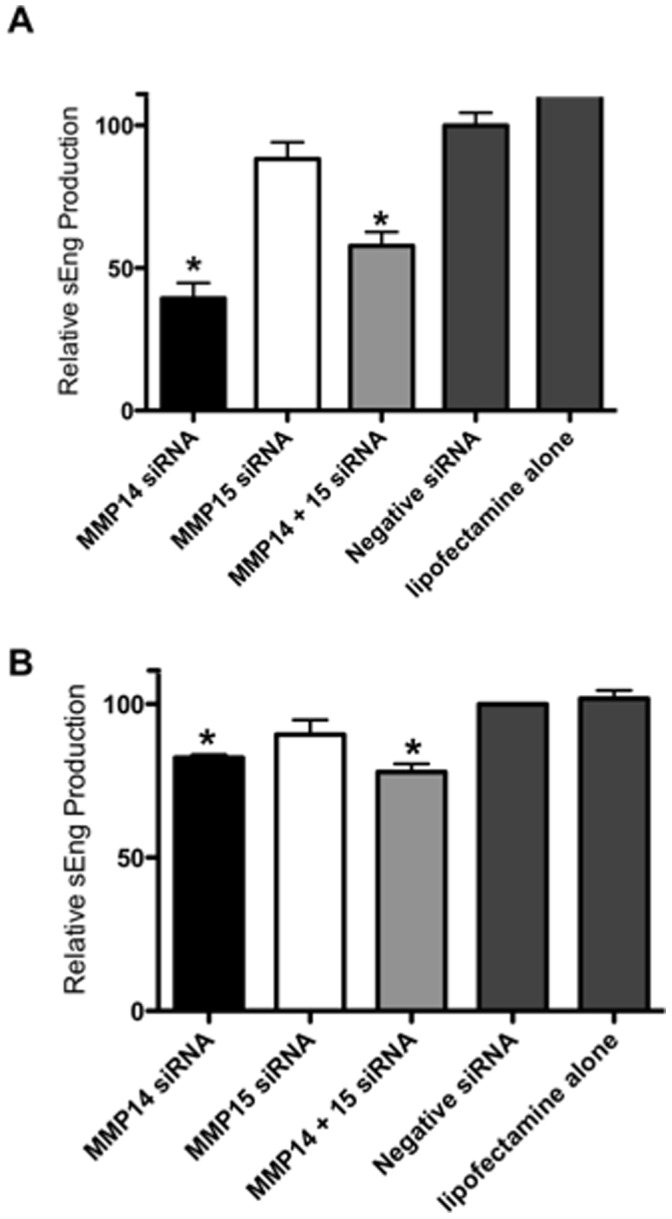
MMP-15 inhibtion does not decrease soluble endoglin production *in vitro*. Treatment of HUVEC cells (**A)** and syncytialised BeWo cells (**B**) with MMP-14 siRNA alone or in combination with MMP-15 siRNA induced a significant decline in sEng production compared to scrambled siRNA, whilst MMP-15 siRNA alone had no effect. Data shown as mean±SEM, n = 3 experiments, *p≤0.05.

### siRNA Knockdown of MMP-14 and MMP-15 *in vitro*

For siRNA experiments, both human umbilical vein endothelial cells (HUVEC – cell line) and syncytialised BeWo cells were used. HUVECs were transfected with either 10 nM MMP-14 siRNA (Qiagen), MMP-15 siRNA (Qiagen), combined MMP-14+ MMP-15 siRNA, negative control siRNA (Qiagen) or transfection reagent alone (Lipofectamine RNAiMAX, Invitrogen). Treatments were applied for 48 h before media was changed and collected 24 h later for endoglin ELISA and cell lysates were collected for mRNA extraction.

BeWo cells were first syncytialised with 20 uM forskolin and left for 48 h. Thereafter they were treated with 40 nM siRNAs as described for HUVECs above. Treatments were applied for 48 h before media was collected for endoglin ELISA and cell lysates were collected for mRNA extraction. All experiments were repeated a minimum of 3 times with at least 4 replicates per treatment.

### RT-PCR

To assess the efficiency of siRNA knockdown, RNA was extracted from HUVEC or BeWo lysates using RNeasy mini kit (Qiagen, Valencia, CA). 0.2 µg of RNA was then converted to cDNA using SuperScript III (Invitrogen) and random hexamers (Invitrogen) as per manufacturers guidelines. Taqman gene expression assays for MMP-14, MMP-15 and GAPDH were used (Applied Biosystems, Carlsbad, CA). RT-PCR was performed on the CFX 384 (Biorad, Hercules, CA) using FAM-labelled Taqman universal PCR mastermix (Applied Biosystems) with the following run conditions: 50°C for 2 minutes; 95°C for 10 mins; 95°C for 15 seconds and 60°C for 1 minute (40 cycles). PCR product was confirmed by gel electrophoresis. Relative quantification was determined using the comparative CT method.

### Statistical Analysis

Continuous variables were compared using either an unpaired t-test to assess parametric data or a Mann Whitney U for non-parametric data. Categorical values were compared using the Chi-squared test. P≤0.05 was considered significant. All statistical analysis was undertaken using GraphPad Prism (GraphPad Software, La Jolla, CA).

## Results

### MMP-15 is Localized to the Syncytiotrophoblast and Up-regulated in Preeclampsia

We first examined expression of MMP-15 in severe, preterm pre-eclamptic placentas and preterm controls. Given soluble endoglin has been implicated in severe preeclampsia, we only examined placentas obtained from cases of very severe disease. Thus, in our entire preeclamptic cohort, iatrogenic preterm delivery was required (<34 weeks gestation) for maternal or fetal indications. The preterm controls were cases of either spontaneous onset of labour or premature rupture of membranes where there was no evidence of maternal infection or hypertensive disease. All samples were collected by caesarean section.

We first assessed endoglin by immunohistochemistry and confirmed it was localized to the syncytiotrophoblast in both pre-eclamptic and pre-term placenta (Figure 1 A, B), consistent with our previous report [12]. We then stained MMP-15 by immunohistochemistry and also found that it localized to the syncytiotrophoblast in both pre-eclamptic and pre-term placenta (Figure 1 D, E). Importantly, immunohistochemistry for MMP-15 and endoglin on serially sectioned placenta indicated co-localisation of the two proteins within the syncytiotrophoblast layer (Figure 1G,H).

Next, we measured expression of MMP-15 in placenta from women with severe early-onset preeclampsia (n = 8) and gestationally matched preterm controls (n = 8). Western analysis showed MMP-15 was increased in preeclamptic placentas compared to preterm controls (Figure 1I,J; p = 0.03).

### MMP-15 Knockdown does not Reduce soluble Endoglin Production by HUVECs or Syncytialised BeWos

We next investigated whether inhibiting MMP-15 using siRNAs would decrease soluble endoglin production *in vitro* using syncytialised BeWos. This cell line best models the syncytiotrophoblast, and we have previously screened a number of placental cell lines and found syncytialised Bewos to be the highest producer of soluble endoglin [12].

Of all tissues in the body, endoglin is most highly expressed in placenta and endothelial cells [14]. Therefore, we also examined the effects of MMP-15 inhibition in HUVECs where we also knocked down MMP-14.

We first confirmed siMMP-14, 15, alone, or 14 and 15 in combination resulted in >85% knockdown compared to negative siRNA in HUVEC cells. In syncytialised BeWos MMP-14 siRNA yielded a mean mRNA knockdown of 35.5±3.9%, whilst MMP-15 siRNA yielded a 77.4±4.2% knockdown compared to negative siRNA. Similar knockdown efficiency was observed when both siRNAs were added in combination.

In HUVEC cells, MMP-14 siRNA significantly decreased sEng by 61±5.5% (p<0.0001 compared to non-targeting siRNA controls), MMP-14 and MMP-15 siRNA in combination induced a 42±4.9% decrease in sEng (p<0.0001), whilst MMP-15 siRNA alone caused no significant change in sEng compared to negative siRNA (Figure 2A). In syncytialised BeWo cells, MMP-14 siRNA significantly (p<0.05) decreased sEng by 18.5±1.0% when transfected alone, whilst combination MMP14+ MMP15 siRNA significantly reduced (p<0.05) sEng production by 22.1±2.6%. No significant change in sEng levels was detected following MMP-15 knockdown alone (Figure 2B). Together these data indicate that MMP-15 does not cleave endoglin to produce soluble endoglin in either endothelial or placental cells, the two tissues types that exhibit the highest expression of endoglin of all tissues in the body.

## Discussion

We recently demonstrated that MMP-14 is the cleavage protease responsible for soluble endoglin release from placenta. However, in those functional studies where we used placental cells and both *in vitro* and *in vivo* models, we were only able to partially decrease sEng release. This suggested other unidentified proteases might also have a role in producing this anti-angiogenic factor.

We therefore undertook this current study to examine whether MMP-15 might be such a protease given its homology to MMP-14 [13], its high placental expression [17] and the fact that both MMP-14 and 15 have recently been shown to have interchangeable roles for placental labyrinth formation and development in mice [15]. In that study where knock-out mice were used, MMP-15 was able to entirely compensate for the absence of MMP-14 with severe phenotypic effects only observed when both were deleted.

The MMP family consists of 24 zinc-dependent endopeptidases. MMP-15, is one of six membrane-type MMPs, which are further classified based upon their cell surface association: MT1, 2, 3 and 5 (also known as MMP-14, 15, 16 and 24) have a transmembrane domain, whilst MT4 and 6 (also known as MMP-17 and MMP-25) are glycophosphatidylinositol anchored. [7], [8], [9], [10].

MMP-15 is expressed in a variety of human tissues including leukocytes, endothelial cells, hepatocytes and placenta [17], [18], [19], [20]. It cleaves gelatin and can degrade a wide range of extracellular matrix molecules including fibronectin and aggrecan [21]. Like MMP-14, MMP-15 has been most widely studied for its role in cancer progression and metastasis, primarily through its capacity to cleave and activate MMP-2 [22], [23], [24]. In this study we have shown MMP-15 is localized to the syncytiotrophoblast and is up-regulated in preeclamptic placentas. We can only speculate on the possible role, if any, that MMP-15 may play in the pathogenesis of preeclampsia. It may be possible that aberrant up-regulation of MMP-15 in preeclampsia may be responsible for the activation of MMP-2, which has also been reported to be upregulated in pre-eclampsia and may contribute to endothelial dysfunction [25].

However, our functional studies suggest MMP-15 is not involved the production of soluble endoglin from either placental or endothelial cells. In both BeWo and HUVEC cells MMP-15 targeted siRNA knockdown produced no significant decline in sEng production compared to control siRNA. Concurrent knockdown of both MMP-14 and 15 did not reduce sEng significantly more than MMP-14 silencing alone. The fact sEng continues to be produced both in this series of experiments and in our previous report that focused on MMP-14 [12] suggests a further mechanism of sEng production exists and awaits characterization.
